# Supplementary material for: Identification of the factor XII contact activation site enables sensitive coagulation diagnostics
Source: Nat Commun. 2021 Sep 22;12:5596. doi: 10.1038/s41467-021-25888-7 (PMC8458485; doi:10.1038/s41467-021-25888-7)
Supplement: Supplementary file 1 — Supplementary Information [file 41467_2021_25888_MOESM1_ESM.pdf]

SUPPLEMENTARY INFORMATION

IDENTIFICATION OF THE FACTOR XII CONTACT ACTIVATION SITE ENABLES SENSITIVE COAGULATION DIAGNOSTICS

Marco Heestermans, Clément Naudin, Reiner K. Mailer, Sandra Konrath, Kristin Klaetschke, Anne Jamsa, Maike Frye, Carsten Deppermann, Giordano Pula, Piotr Kuta, Manuel A. Friese, Mathias Gelderblom, Albert Sickmann, Roger J. S. Preston, Jerzy-Roch Nofer, Stefan Rose-John, Lynn M. Butler, Ophira Salomon, Evi X. Stavrou, and Thomas Renné

SUPPLEMENTARY TABLE 1. Primers used for the generation of FXII mutants.

Numbering from *F12* mRNA; (OMIM ID: 610619 Sequence).

Primer name	Sequence (5’ to 3’)
FXII_EcoRI-Fib-II	^{146}GCTGAAGAGCACACAGTCGAATTCACTGTCACCGGGGAGC^{185}
FXII_EcoRI-EGF-I	^{299}CGATGGGGATACTGTTTGGAATTCAAGAAAGTGAAAGACC^{338}
FXII_EcoRI-Fib-I	^{429}GAAACCACTGCCAGGAATTCAAGTGCTTTGAGC^{461}
FXII_EcoRI-EGF-II_1	^{553}CCACTGCCAGCGGAATTCAGCCAGGCCTGCC^{585}
FXII_EcorRI-EGF-II_2	^{605}GGGGGTCGCTGCCTAGAATTCGAGGGCCACCGCCTG^{640}
FXII_EcoRI-Kringle	^{670}CTTCTGCGACGTGGAATTCAAGGCAAGCTGCT^{701}
FXII_EcoRI-PR-I	^{922}CCTGGCACAGTGCGAATTCCCAACCCAGGCGG^{953}
FXII_EcoRI-PR-II	^{966}CGGTGTCCCCTAGGCTTGAATTCCCACTCATGCCCGCGC^{1005}
FXII_EcoRI-PR-III	^{1034}ACCCGGACCCCGCCTGAATTCCAGACCCCGGGAGCC^{1069}
FXII_EcoRI-PR_C-term	^{1111}CGGCCCACTGAGCGAATTCCAGCGGCTCCGCA^{1142}
FXII_EcoRI-LC	^{1148}CTGTCTTCGATGACCGAATTCGTTGGCGGGCTGGTG^{1183}

SUPPLEMENTARY FIGURES

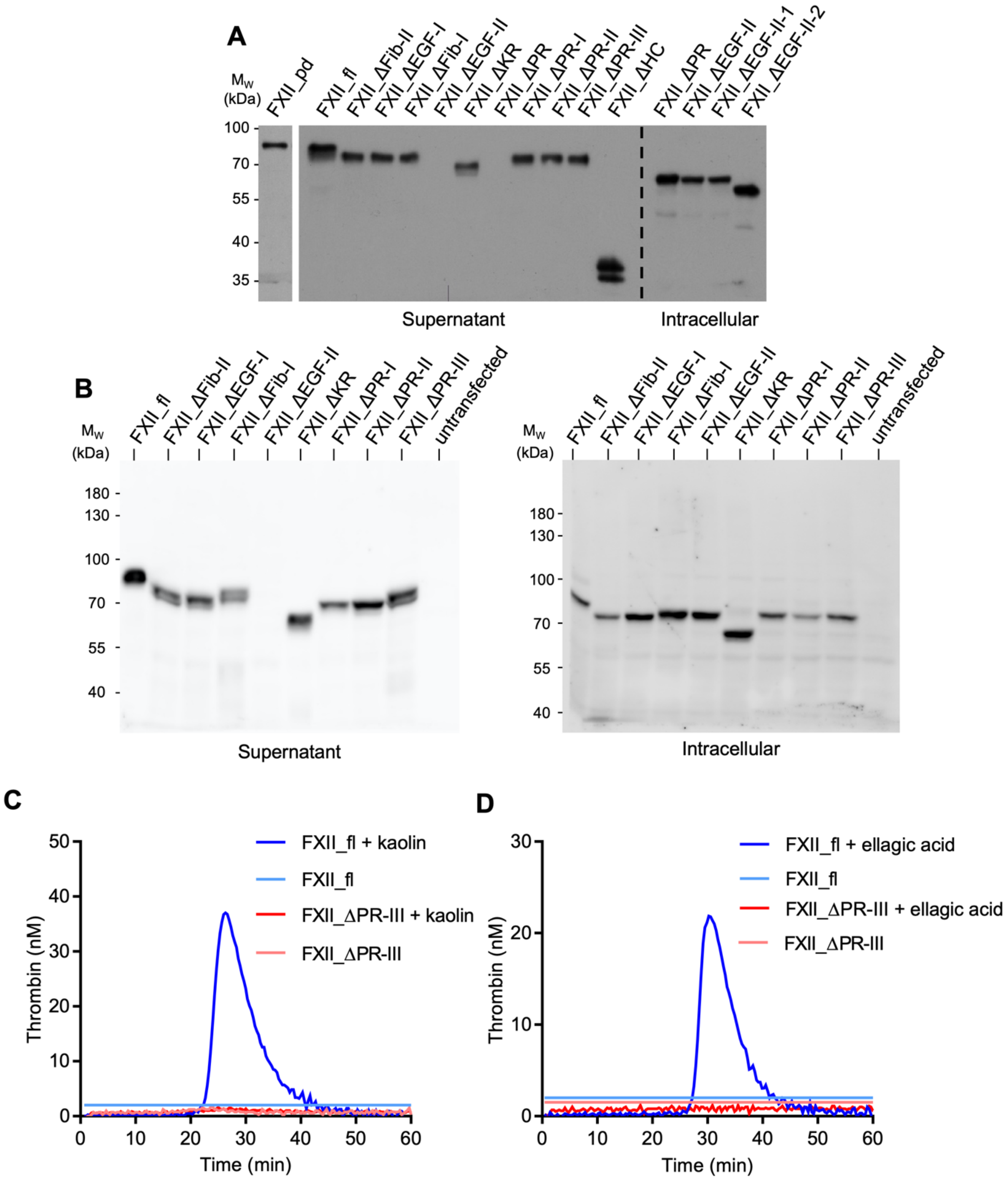

SUPPLEMENTARY FIGURE 1: Expression and characterization of FXII mutants in HepG2 and CHO cells. (A, B) HepG2 (A) or CHO-K1 (B) cells were transiently transfected with vectors coding for FXII mutants shown in Figure 1A. 48 h after transfection, washed cells or cell supernatants were analyzed for FXII protein using SDS-PAGE under reducing conditions and by western immunoblotting with polyclonal anti-FXII antibodies. Representative images of n=3 individual experiments. (C, D) Real time thrombin generation curves of human FXII-deficient plasma reconstituted with CHO-expressed FXII_fl or FXII_ΔPR-III, prior to activation with kaolin (C) or ellagic acid (D). n=3 separate experiments run in duplicate each. Pd: plasma-derived, fl: full length, Fib-II: fibronectin type-II domain, EGF-I: the first epidermal growth factor-like domain, Fib-I: fibronectin type-I domain, EGF-II: the second EGF-like domain, KR: Kringle, PR: proline-rich domains, HC: heavy chain. M_w: molecular weight, kDa: kilodalton.

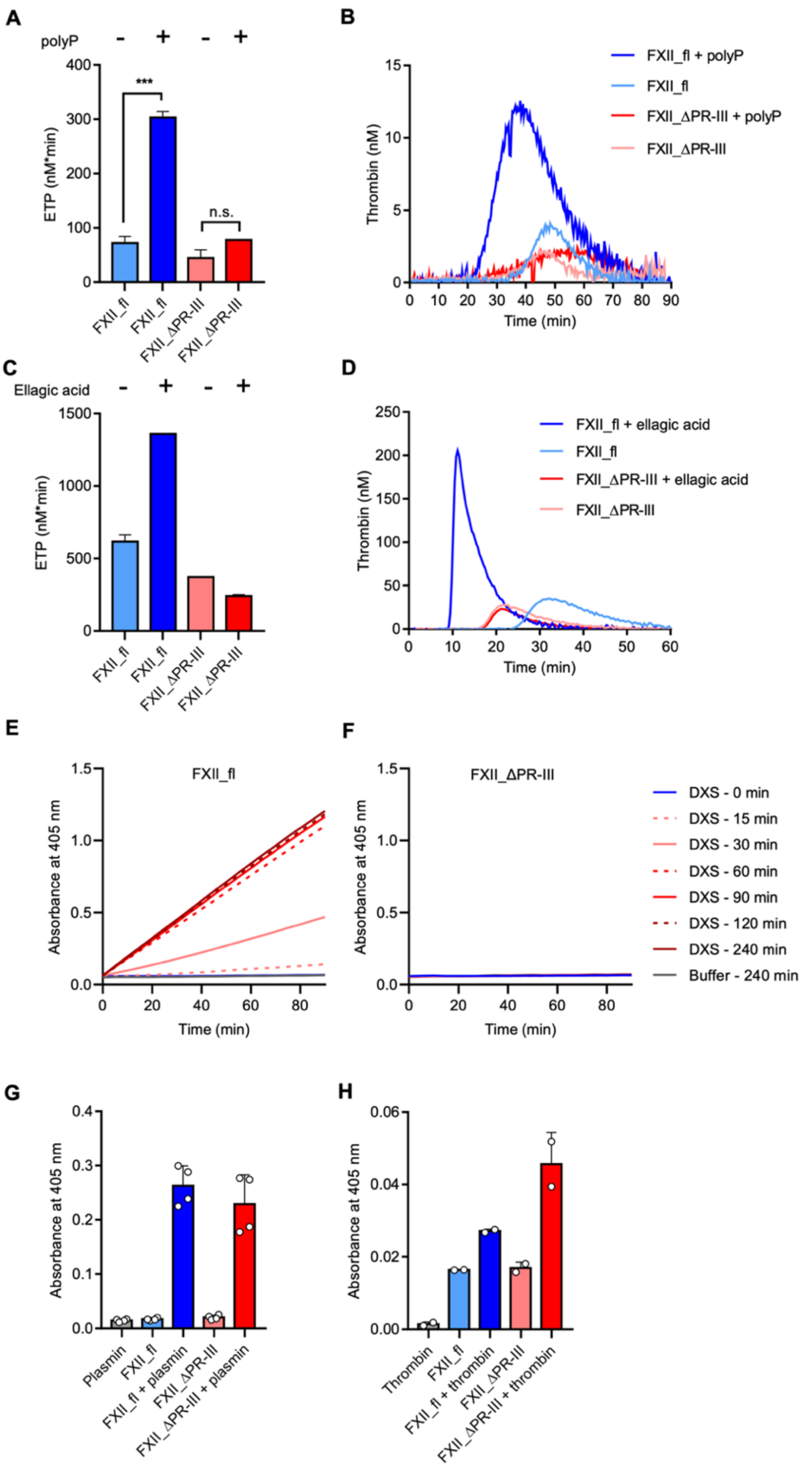

SUPPLEMENTARY FIGURE 2: Defective contact activation of FXII_ΔPR-III. (A-D) Real time thrombin generation was performed with FXII-deficient plasma, spiked with FXII_fl or FXII_ΔPR-III. (A, C) ETP of samples stimulated with (A) *E. coli* polyP (+, 10 µg/mL), (C) ellagic acid (+, 2.5 µg/mL) or buffer (-), n=3 individual experiments, each run in triplicate. n.s.: non-significant, ***: P<0.001, by unpaired two-tailed Student´s t test. (B, D) Representative real time thrombin generation curves of panel A and C, respectively. (E, F) FXII_fl and FXII_ΔPR-III, were incubated with DXS (1 µg/mL) for 0, 15, 30, 60, 90, 120 and 240 min at 37°C. FXIIa formation was measured by the conversion of the chromogenic substrate S2302 at 405 nm for 90 min. Representative images of n=3 individual experiments. (G, H) FXII_fl and FXII_ΔPR-III were activated with plasmin (G, 8 U/L) or thrombin (H, 4 U/L) and formed FXIIa was measured chromogenically at 120 min. n=4 (panel G), n=2 (panel H) individual chromogenic assays. Columns give means ± SD.

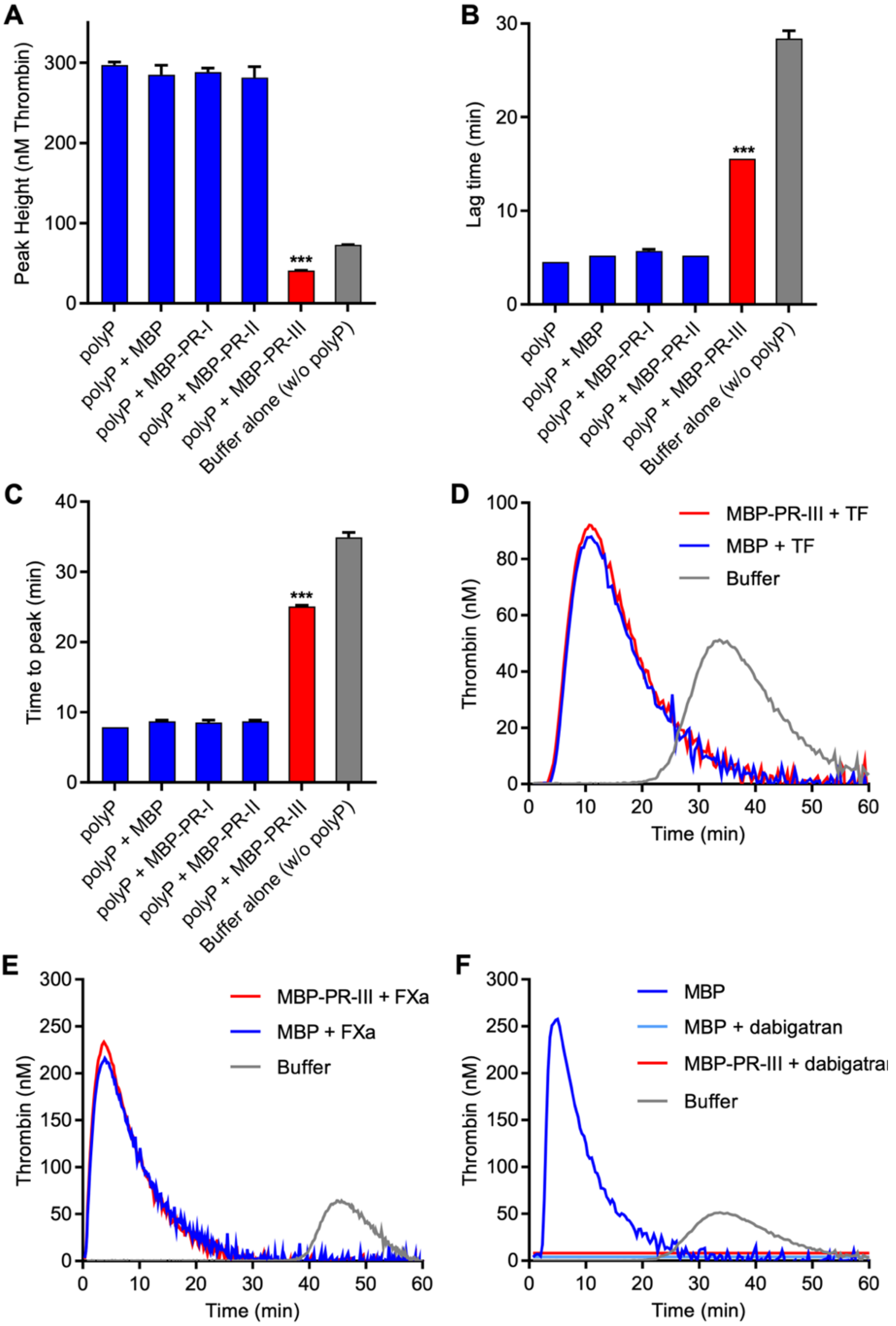

SUPPLEMENTARY FIGURE 3. PR-III specifically interferes with contact activation-triggered thrombin formation. (A-C) Peak thrombin (panel A), lag time (panel B) and time to peak (panel C) in normal PPP stimulated with long-chain polyP (5 µg/mL) following preincubation with MBP, MBP-PR-I, MBP-PR-II or MBP-PR-III (600 nM each) or buffer alone (w/o polyP). Data were obtained from the experiment shown in **Fig. 4, C and D**. n=3 run in triplicate each. ***: P<0.001 compared to polyP alone by one-way ANOVA and Dunnett´s multiple comparison test. Columns present means ± SD. (D - F) Real time thrombin generation in normal PPP stimulated with FXa (E, 0.4 U/ml), TF (F, 1 pM) or long-chain polyP (F, 5 µg/mL) in the presence of MBP-PR-III, MBP (3.5 µM each) or buffer. In experiments shown in F some plasma samples were spiked with dabigatran (700 ng/ml). Experiments were run in triplicate and independently performed four times.

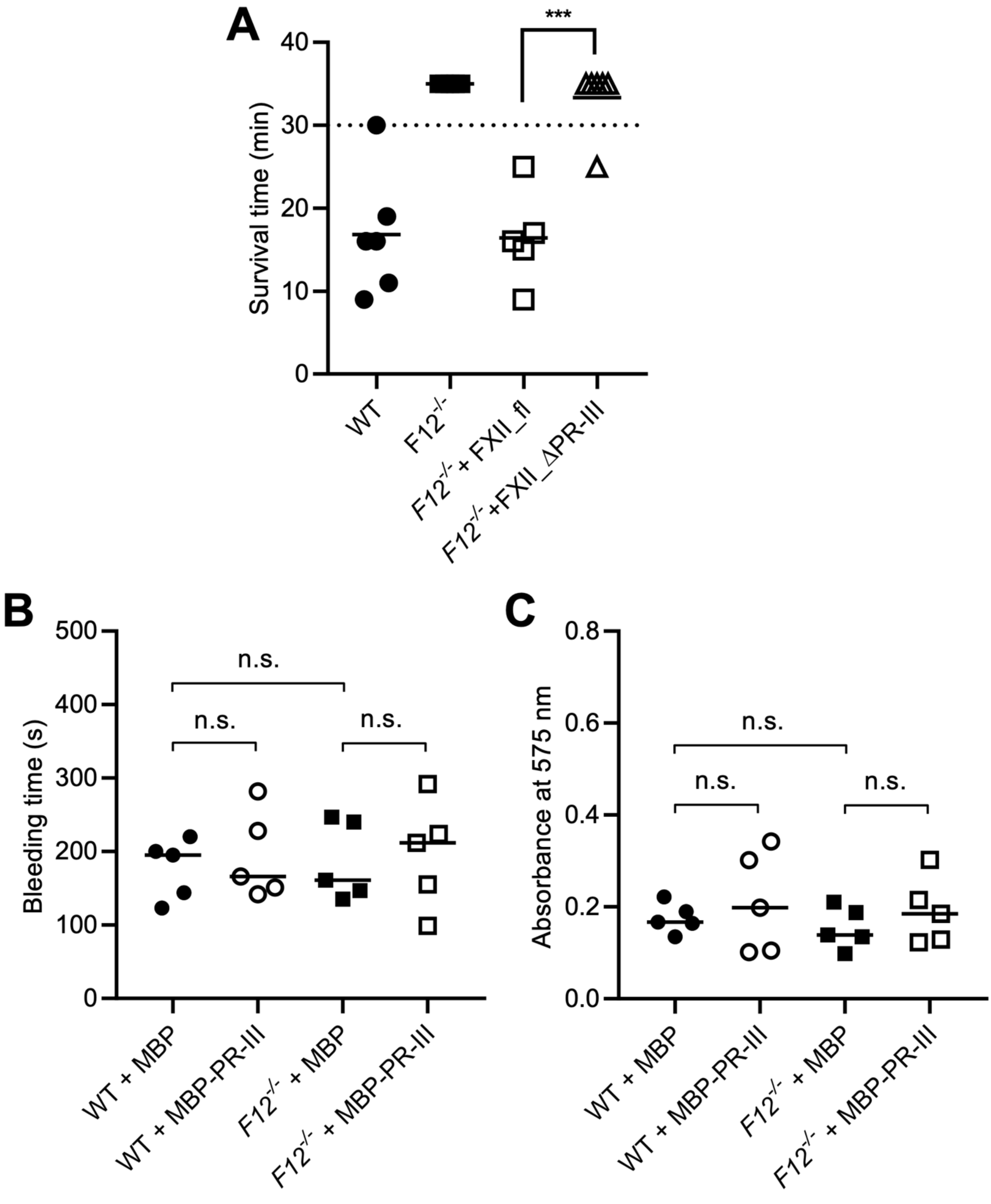

SUPPLEMENTARY FIGURE 4: PR-III influences thrombosis but does not affect hemostasis. (A) Pulmonary embolism was induced by intravenous injection of kaolin (250 µg/g body weight). The survival time of WT, *F12*$^{-/-}$ or *F12*$^{-/-}$ mice reconstituted with recombinant FXII_ΔPR-III or FXII_fl prior to challenge was monitored. Mortality was assessed in each group of mice, and animals alive 30 min after the challenge were considered survivors. n=6 mice. (B, C) Bleeding times and blood loss from clipped tails of WT and *F12*$^{-/-}$ mice, injected with MBP or MBP-PR-III. (B) Bleeding time and (C) total hemoglobin loss was determined by the absorbance of hemoglobin in 37°C phosphate buffered saline, λ=575 nm. n=5 animals. Each symbol represents one animal. Lines show means. A - C: ***: P<0.001, n.s. P>0.05 by unpaired two-tailed Student´s t test. Each symbol represents one individual animal

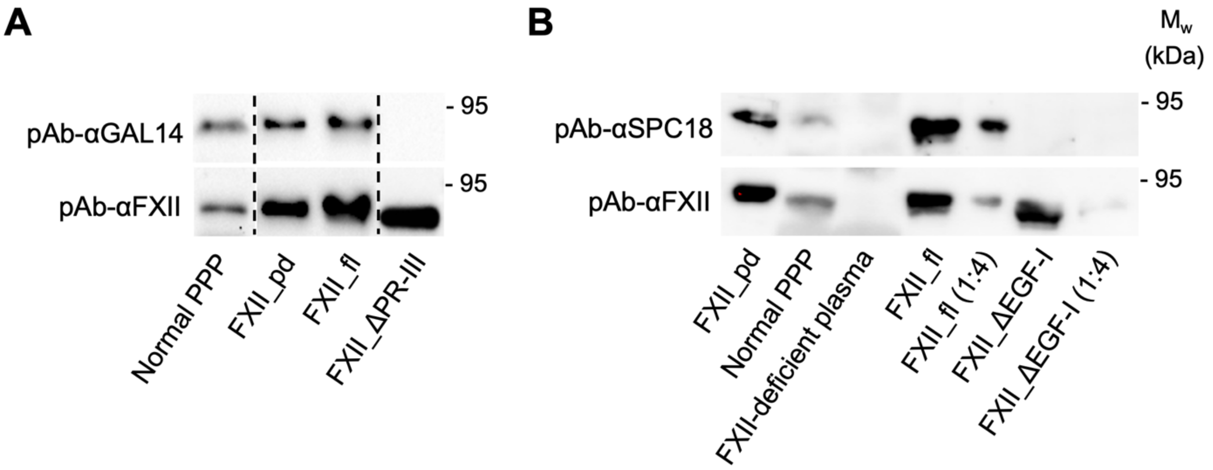

SUPPLEMENTARY FIGURE 5. pAB-αGAL14 and pAB-αSPC18 characterization. Western blotting of normal PPP, FXII_pd, FXII_fl, FXII_ΔPR-III, FXII_ΔEGF-I or 1:4 diluted FXII_fl or FXII_ΔEGF-I using pAB-αGAL14 (A, top panel) or pAB-αSPC18 (B, top panel). For loading control, blotted membranes were also probed with an antibody against the common FXII light chain (pAb-αFXII, bottom panels). Representative western blots of n=2 individual experiments. PPP: platelet poor plasma, pd: plasma derived, fl: full length, PR: proline rich domain. EGF-I: the first epidermal growth factor like domain.

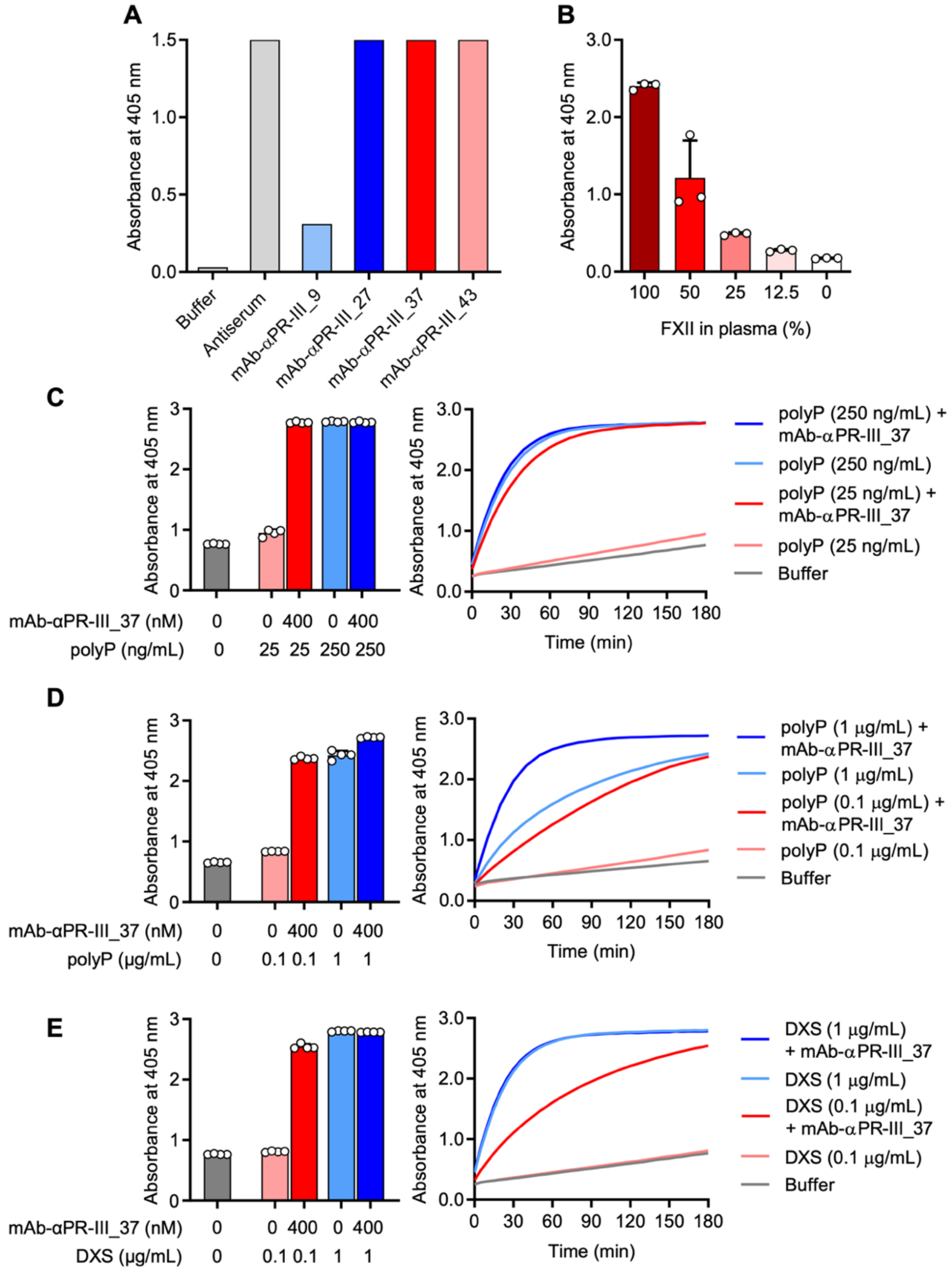

SUPPLEMENTARY FIGURE 6. Characterization of mAb-αPR-III_37 antibody. (A) Binding of anti-PR-III monoclonal antibodies mAb-αPR-III_9, mAb-αPR-III_27, mAb-αPR-III_37 or mAb-αPR-III_43 to immobilized GAL14 peptide in an ELISA. A representative experiment of a series of n=3 is shown. (B) Mixtures of normal PPP and FXII-deficient plasma were incubated for 1 h with mAb-αPR-III_37 (750 nM), after which S2302 was added. FXIIa formation was measured after 180 min. n=3 run in triplicate each. (C-E) Normal PPP was supplemented with buffer or mAb-αPR-III_37 (150 nM, molar ratio 1:1), and subsequently a miniscule (red/pink bars) or high (blue bars) concentration of long-chain polyP (panel C), short-chain polyP (panel D), or dextran sulfate (DXS, panel E) was added. FXIIa formation was measured using S2302 conversion and is shown at 180 min by column bars. n=4, experiments independently performed three times. Columns present means ± SD

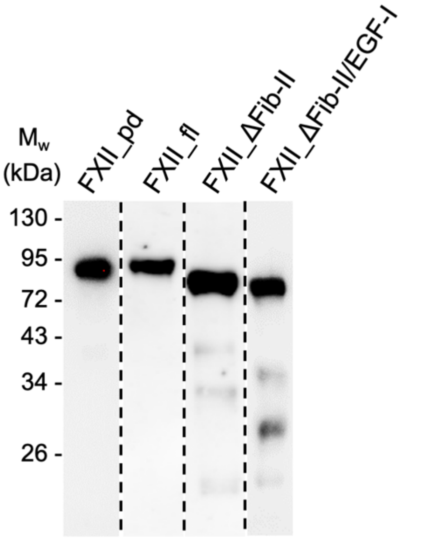

SUPPLEMENTARY FIGURE 7. Instability of FXII_ΔFib-II and FXII_ΔFib-II/EGF-I mutant proteins. Supernatants of FXII_fl, FXII_ΔFib-II and FXII_ΔFib-II/EGF-I expressed in HEK293 cells were probed with a polyclonal anti-FXII antibody in immunoprint analyses. Pure FXII_pd was loaded for comparison. A representative blot of n=4 is shown. Pd: plasma derived, fl: full length, Fib-II: fibronectin type-II domain, EGF-I: the first epidermal growth factor like domain.

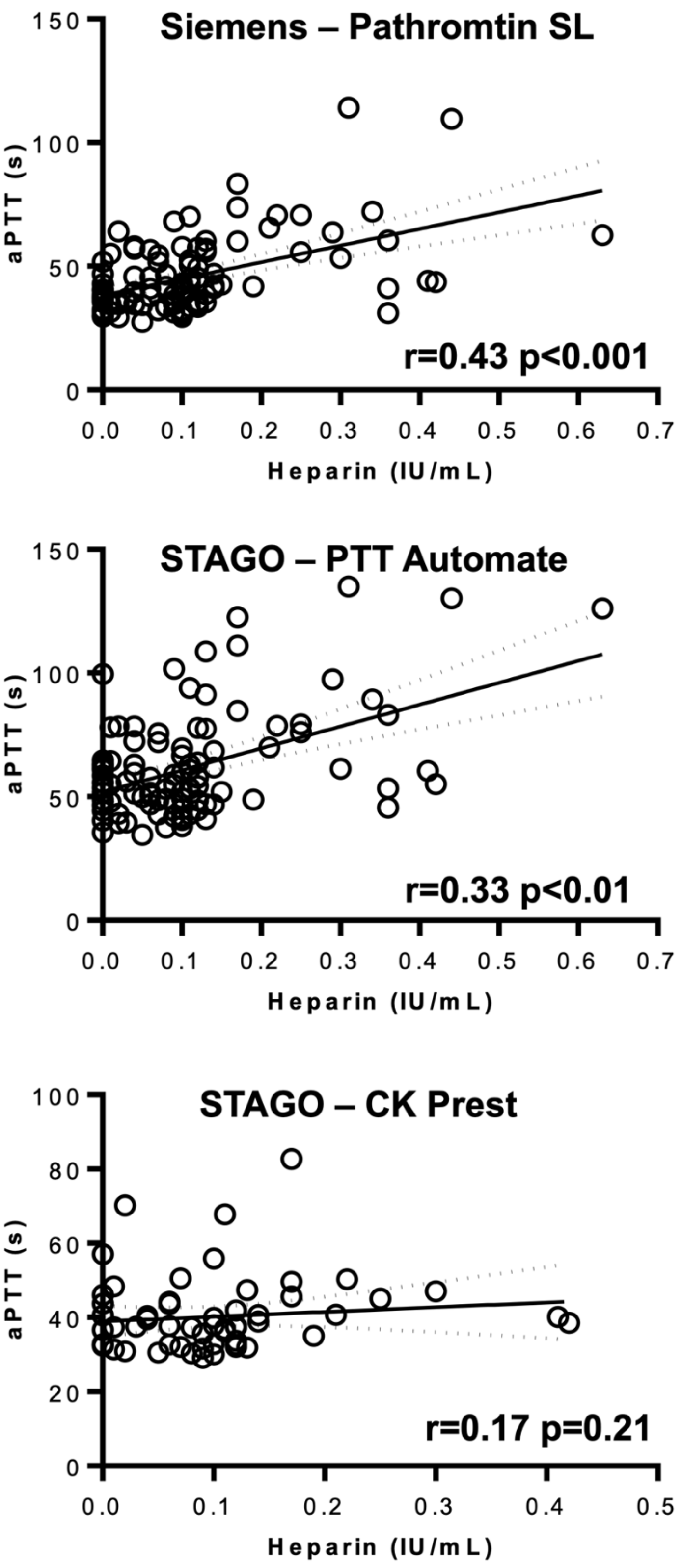

SUPPLEMENTARY FIGURE 8. Particulate reagent-driven aPTT assays have low sensitivity in capturing the anticoagulant activity of UFH. Platelet poor plasma samples were obtained from intensive care patients receiving therapeutic doses of intravenous unfractionated heparin (UFH) and who were monitored by an aPTT assay performed on a Siemens BCS-XP or STAGO StarMax3 analyzer, using (A) Siemens Pathromtin SL (n=121), (B) Stago PTT Automate (n=119) and (C) Stago CK Prest (n=78) particle-based reagents. Heparin concentrations were measured using the chromogenic Innovance Heparin Assay from Siemens.
