# Supplementary material for: Identification of the factor XII contact activation site enables sensitive coagulation diagnostics
Source: Nat Commun. 2021 Sep 22;12:5596. doi: 10.1038/s41467-021-25888-7 (PMC8458485; doi:10.1038/s41467-021-25888-7)
Supplement: Supplementary file 3 — Reporting Summary [file 41467_2021_25888_MOESM3_ESM.pdf]

nature research

Corresponding author(s): Thomas Renné

Last updated by author(s): Jul 27, 2021

Reporting Summary

Nature Research wishes to improve the reproducibility of the work that we publish. This form provides structure for consistency and transparency in reporting. For further information on Nature Research policies, see our Editorial Policies and the Editorial Policy Checklist.

Statistics

For all statistical analyses, confirm that the following items are present in the figure legend, table legend, main text, or Methods section.

n/a	Confirmed	
☐	☒	The exact sample size (n) for each experimental group/condition, given as a discrete number and unit of measurement
☐	☒	A statement on whether measurements were taken from distinct samples or whether the same sample was measured repeatedly
☐	☒	The statistical test(s) used AND whether they are one- or two-sided<br>*Only common tests should be described solely by name; describe more complex techniques in the Methods section.*
☒	☐	A description of all covariates tested
☒	☐	A description of any assumptions or corrections, such as tests of normality and adjustment for multiple comparisons
☐	☒	A full description of the statistical parameters including central tendency (e.g. means) or other basic estimates (e.g. regression coefficient) AND variation (e.g. standard deviation) or associated estimates of uncertainty (e.g. confidence intervals)
☐	☒	For null hypothesis testing, the test statistic (e.g. F, t, r) with confidence intervals, effect sizes, degrees of freedom and P value noted<br>*Give P values as exact values whenever suitable.*
☒	☐	For Bayesian analysis, information on the choice of priors and Markov chain Monte Carlo settings
☒	☐	For hierarchical and complex designs, identification of the appropriate level for tests and full reporting of outcomes
☒	☐	Estimates of effect sizes (e.g. Cohen's d, Pearson's r), indicating how they were calculated

Our web collection on statistics for biologists contains articles on many of the points above.

Software and code

Policy information about availability of computer code

Data collection	Thrombinoscope software package (Version 3.0.0.29); Prism 8 for macOS, Version 8.4.3
Data analysis	Prism 8 for macOS, Version 8.4.3

For manuscripts utilizing custom algorithms or software that are central to the research but not yet described in published literature, software must be made available to editors and reviewers. We strongly encourage code deposition in a community repository (e.g. GitHub). See the Nature Research guidelines for submitting code & software for further information.

Data

Policy information about availability of data

All manuscripts must include a data availability statement. This statement should provide the following information, where applicable:

- Accession codes, unique identifiers, or web links for publicly available datasets
- A list of figures that have associated raw data
- A description of any restrictions on data availability

DATA AVAILABILITY
The authors declare that the data supporting the findings of this study are available within the article and from the authors upon request.

Field-specific reporting

Please select the one below that is the best fit for your research. If you are not sure, read the appropriate sections before making your selection.

☒ Life sciences ☐ Behavioural & social sciences ☐ Ecological, evolutionary & environmental sciences

For a reference copy of the document with all sections, see nature.com/documents/nr-reporting-summary-flat.pdf

Life sciences study design

All studies must disclose on these points even when the disclosure is negative.

Sample size	Sample or experiment sizes were determined empirically based on previous experience, e.g. Larsson M et al. Sci Trans Med 2014; no statistical tests were used to predetermine the size of the experiments. FXII zymogen contact activation proceeds by a PKa mediated amplification loop. This specific mechanisms with internal amplification either results in (almost) complete or no detectable activation. Therefore a gradual or partial activation is not common and the sample size can be rather limited as we essentially find activation or no activation without grading. To check if data were normally distributed, a quantile-quantile plot was used and data were analyzed by Student's t test or, in the case of multiple comparisons, one-way analysis of variance (ANOVA) followed by post hoc analysis using Dunnett's multiple comparisons test.
Data exclusions	All data for all experiments were included. In previous versions of the manuscript prior to revision we had excluded an outlier, however this experiment and the corresponding data set is not shown in the final manuscript.
Replication	Key experiments were performed and repeated from independent researchers over several years. Data was obtained in three distinct laboratories (Würzburg, Stockholm, Hamburg) with highly similar results. Key experiments were performed from two researchers independently, in a blinded fashion. Attempts for replications were all successful. We controlled quality of coagulation tests by inclusions of samples & materials that are used in an accreditated clinical diagnostic laboratory. Number of replications was based on our established experience with coagulantion assays ex vivo and in mice. The n-number of the experiment shown is given in the legend. Coagulation experiments, competition assays and thrombosis models were performed multiple times (n of 2- 21) with comparable results. Our study design comprises three complementary approaches: 1. deletion mutants, 2. competition studies, 3. polyclonal and monoclonal antibodies. These independent approaches come to the same conclusion and show a critical fole of PR-III for FXII contact activation.
Randomization	Animals were randomly allocated to challenges. The coagulation tests used in the study contain positive and negative controls and are highly standardized and quality controlled and do not require randomization. Factor deficient plasma from different lots and distinct sources was used as indicated in the material section. For the activity assays using chromogenic substrates allocation nor randomization is required as postive / negative controls were included and importantly findings were confirmed by independent assays (real time thrombin formation and/ or clotting).
Blinding	Observers were blinded to animal treatments, and otherwise blinding was performed for data analyses. For experiments other than animal experiments, blinding was not relevant to data collection as calibrated coagulation analyzers (Kugelkoagulometer and CAT with internal controls/standard) were used. The real time thrombin analyses were automatically recorded by the machine and means +/- SD was directly reported without calculations by the researcher. As described above the complementary study design (mutants, competition experiments and various antibodies) offers independent internal controls.

Reporting for specific materials, systems and methods

We require information from authors about some types of materials, experimental systems and methods used in many studies. Here, indicate whether each material, system or method listed is relevant to your study. If you are not sure if a list item applies to your research, read the appropriate section before selecting a response.

Materials & experimental systems

n/a	Involved in the study	
☐	☒	Antibodies
☐	☒	Eukaryotic cell lines
☒	☐	Palaeontology and archaeology
☐	☒	Animals and other organisms
☒	☐	Human research participants
☒	☐	Clinical data
☒	☐	Dual use research of concern

Methods

n/a	Involved in the study	
☒	☐	ChIP-seq
☒	☐	Flow cytometry
☒	☐	MRI-based neuroimaging

Antibodies

Antibodies used	Anti FXII antibodies were obtained from Nordic MUbio (cat. number GAHu/FXII), page 17 of the manuscript. Anti-MBP antibodies were a kind gift from Dr. Werner Müller Esterl, Frankfurt, and raises against recombinant MBP in rabbilts. Anti-MBP antibodies were desribed by Dr. Werner Müller Esterl, in the reference 63 (Herwald H et al, J Biol Chem 1996) on page 16. HRP-coupled donkey anti-rabbit antibody was purchased from Jackson Immunoresearch, AB_2340585 (page 16).

Validation	Primary antibodies were validated using (i) plasma samples with inherited deficiency in the specific antigen, (ii) overexpression systems and (iii) comparisons with other antibodies against the same antigen.<br>Specifically: anti-FXII antibody failed to detect a signal in plasma with inherited FXII deficiency and probed for proteolysis froducts follwing FXII contact activation (Björkqvist J et al, J Clin Invest 2015)<br>Specificity of anti-MBP antibody has been conformed previously (Herwald H et al, J Biol Chem 1996). In our hands, anti-MBP failed to give a signal prior to IPTG-mediated stimulation of MBP-fusion protein expression.<br>Specificity of HRP-coupled donkey anti-rabbit antibody is confirmed by Jackson Immunoresearch. Furthermore, we obtained similar results using an other HRP-coupled donkey anti-rabbit antibody obtained by DAKO company.

Eukaryotic cell lines

Policy information about cell lines

Cell line source(s)	HEK293 (ATCC: CRL-3216), HepG2 (ATCC: BT-8065) or CHO-K1 (ATCC: CCL-61)
Authentication	the three typical immortalized cell lines described above were from commercial sources. Cell lines used were not authenticated since we used them for recombinant protein expression and not for cell type specific assays (e.g signalling).
Mycoplasma contamination	All cells were tested negatively
Commonly misidentified lines<br>(See ICLAC register)	No commonly misidentified cell lines were used in the study. We only used the cell lines for recombinant protein expression.

Animals and other organisms

Policy information about studies involving animals; ARRIVE guidelines recommended for reporting animal research

Laboratory animals	FXII deficient (F12-/-) mice were 6-12 weeks of age and of both sexes as originally described (Pauer et al, reference 61) and used in various later studies (e.g. refs. 6, 23, 26) were used for the thrombosis studies (shown in figure 5 and supplemental figure S4). Littermate wild type controls of the same age and sex were used. All work with BALB/c mice and generation of antibodies in BALB/c mice was performed by Biogenes company Berlin (https://www.biogenes.de/custom-antibodies). F12-/- and wild type mice were kept in the animal facility of the University Medical Center Hamburg. Mice were kept with a 12 h light/12 h dark cycle and researchers and technicians did not enter the mouse room during the dark cycle. Temperatures were constantly ~19-21°C with 40-60% humidity.
Wild animals	No wild animals were used in the study.
Field-collected samples	No field collected samples were used in the study
Ethics oversight	Animal experiments are approved by the University Medical Center Hamburg-Eppendorf local authorities (Tierversuchsantrag, TVA #76/16).

Note that full information on the approval of the study protocol must also be provided in the manuscript.
