# Supplementary material for: Identification of the factor XII contact activation site enables sensitive coagulation diagnostics
Source: Nat Commun. 2021 Sep 22;12:5596. doi: 10.1038/s41467-021-25888-7 (PMC8458485; doi:10.1038/s41467-021-25888-7)
Supplement: Supplementary file 4 — Source Data [file 41467_2021_25888_MOESM4_ESM.zip › SOURCE DATA/Supplementary Information/Source Data Supplementary Figure 5.pptx]

## Slide 1
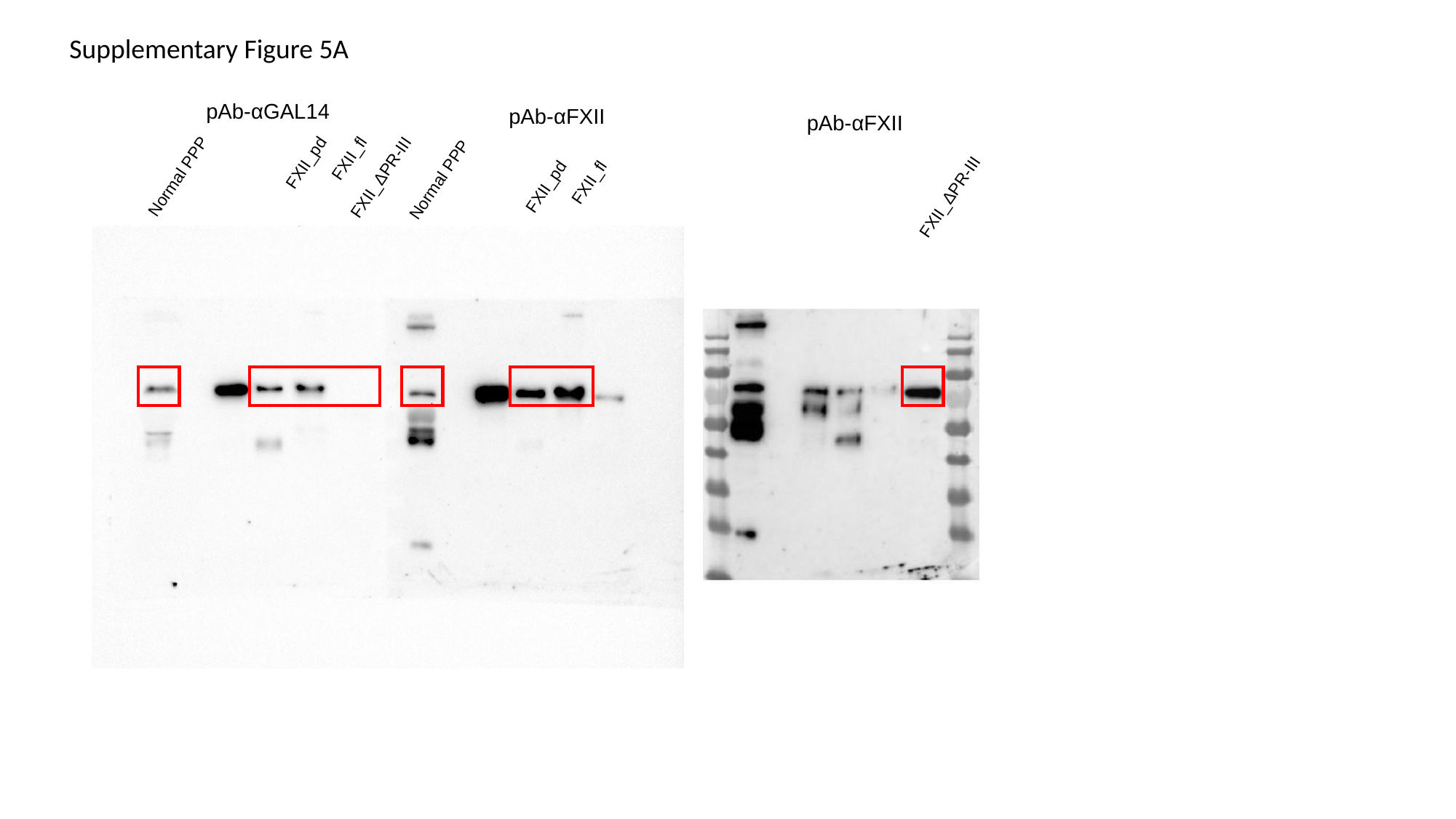

Supplementary Figure 5A
pAb-αGAL14
pAb-αFXII
pAb-αFXII
FXII_ΔPR-III
Normal PPP
FXII_pd
FXII_fl
Normal PPP
FXII_ΔPR-III
FXII_pd
FXII_fl

## Slide 2
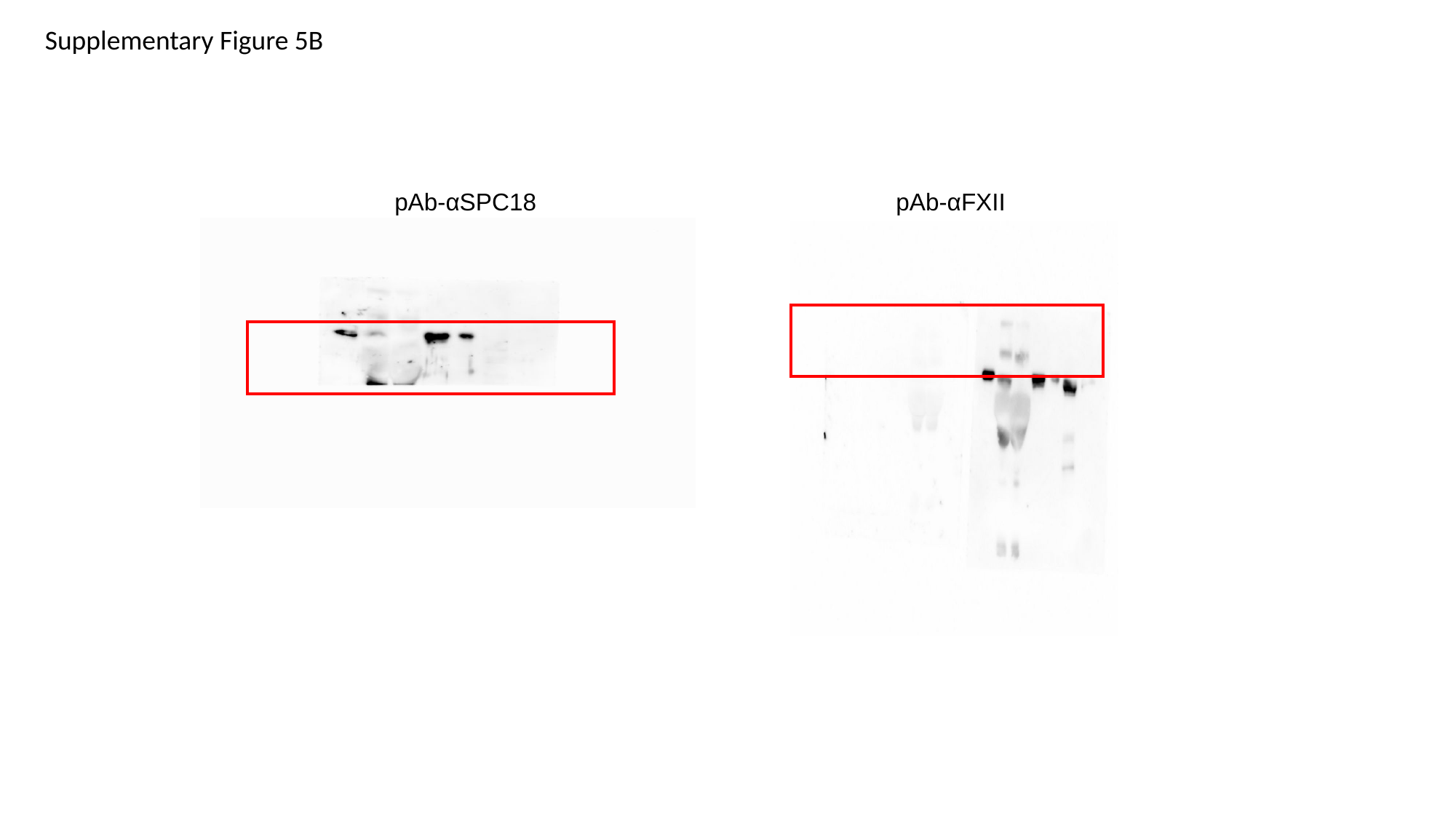

Supplementary Figure 5B
pAb-αSPC18
pAb-αFXII
