# Supplementary material for: Identification of the factor XII contact activation site enables sensitive coagulation diagnostics
Source: Nat Commun. 2021 Sep 22;12:5596. doi: 10.1038/s41467-021-25888-7 (PMC8458485; doi:10.1038/s41467-021-25888-7)
Supplement: Supplementary file 4 — Source Data [file 41467_2021_25888_MOESM4_ESM.zip › SOURCE DATA/Main Manuscript/Source Data 7B_ABclones_western blot.pptx]

## Slide 1
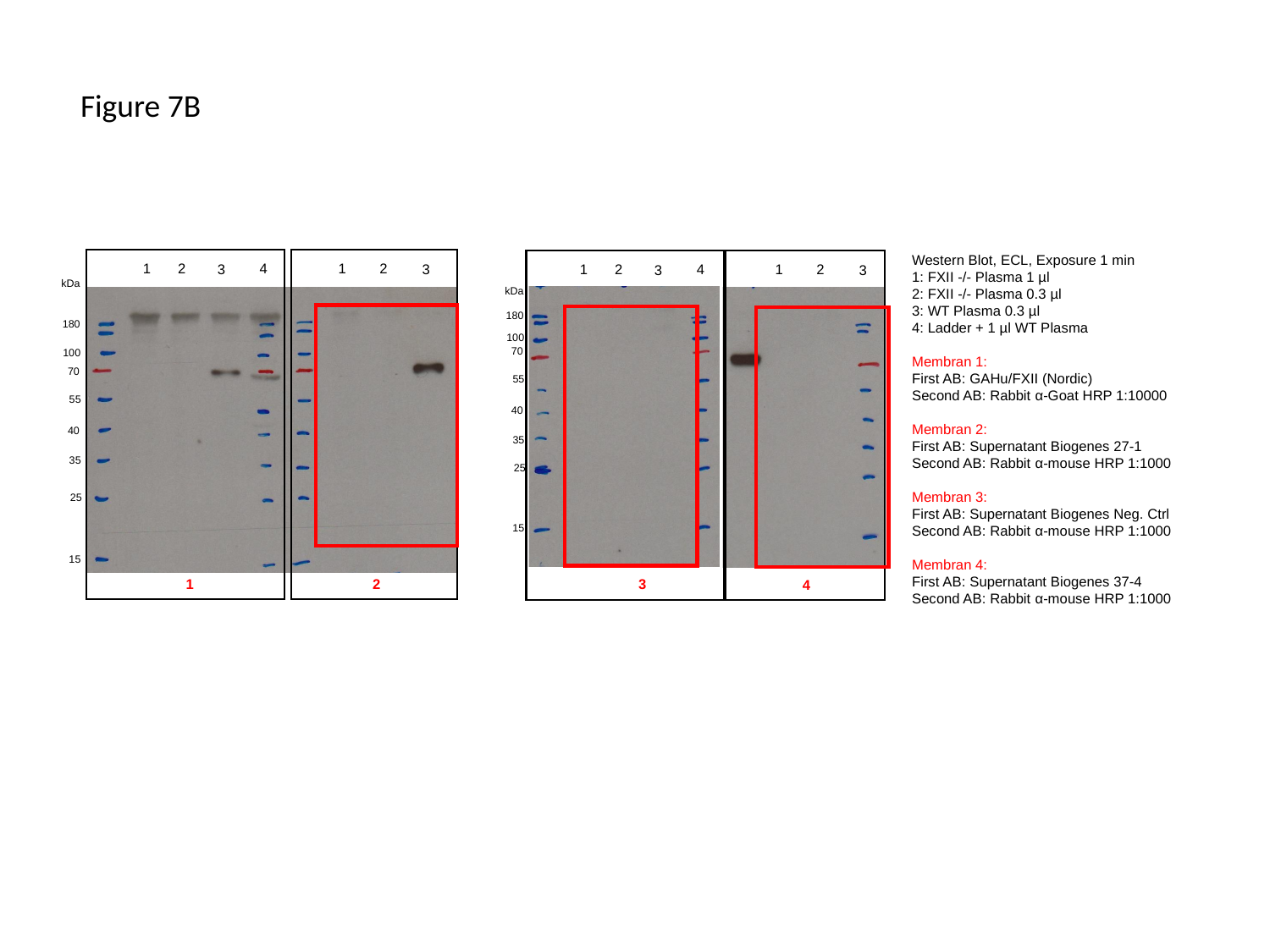

Figure 7B
Western Blot, ECL, Exposure 1 min
1: FXII -/- Plasma 1 µl
2: FXII -/- Plasma 0.3 µl
3: WT Plasma 0.3 µl
4: Ladder + 1 µl WT Plasma
Membran 1:
First AB: GAHu/FXII (Nordic)
Second AB: Rabbit α-Goat HRP 1:10000
Membran 2:
First AB: Supernatant Biogenes 27-1
Second AB: Rabbit α-mouse HRP 1:1000
Membran 3:
First AB: Supernatant Biogenes Neg. Ctrl
Second AB: Rabbit α-mouse HRP 1:1000
Membran 4:
First AB: Supernatant Biogenes 37-4
Second AB: Rabbit α-mouse HRP 1:1000
1
2
1
2
4
3
3
1
2
1
2
4
3
3
kDa
kDa
180
180
100
70
100
70
55
55
40
40
35
35
25
25
15
15
2
3
1
4
