# Supplementary figures and images for: Identification of the factor XII contact activation site enables sensitive coagulation diagnostics
Source: Nat Commun. 2021 Sep 22;12:5596. doi: 10.1038/s41467-021-25888-7 (PMC8458485; doi:10.1038/s41467-021-25888-7)

## Slide 1
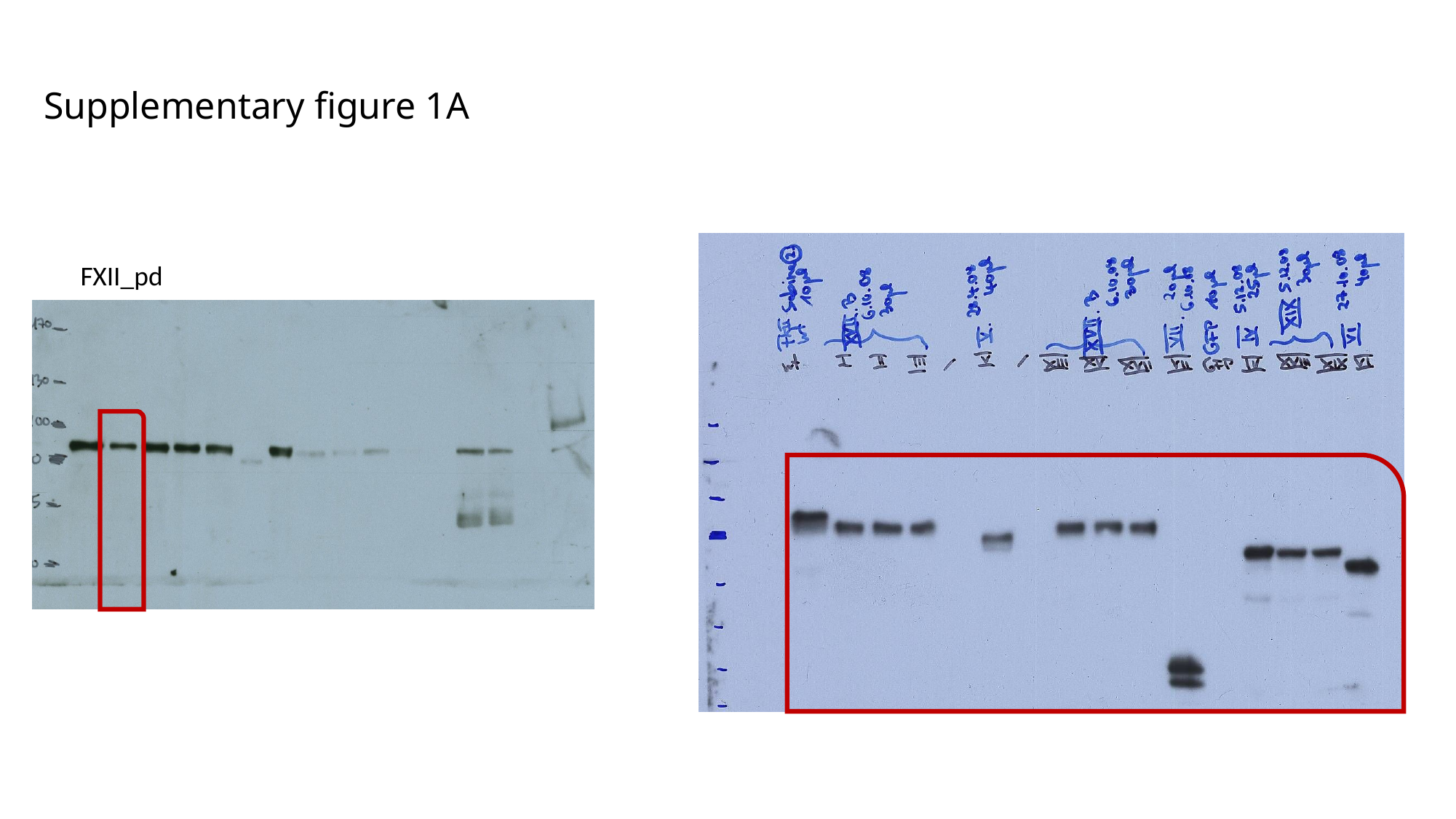

# Supplementary figure 1A
FXII_pd

Supplement: Supplementary file 4 — Source Data [file 41467_2021_25888_MOESM4_ESM.zip › SOURCE DATA/Supplementary Information/Source Data Supplementary Figure 1A.pptx]

## Slide 1
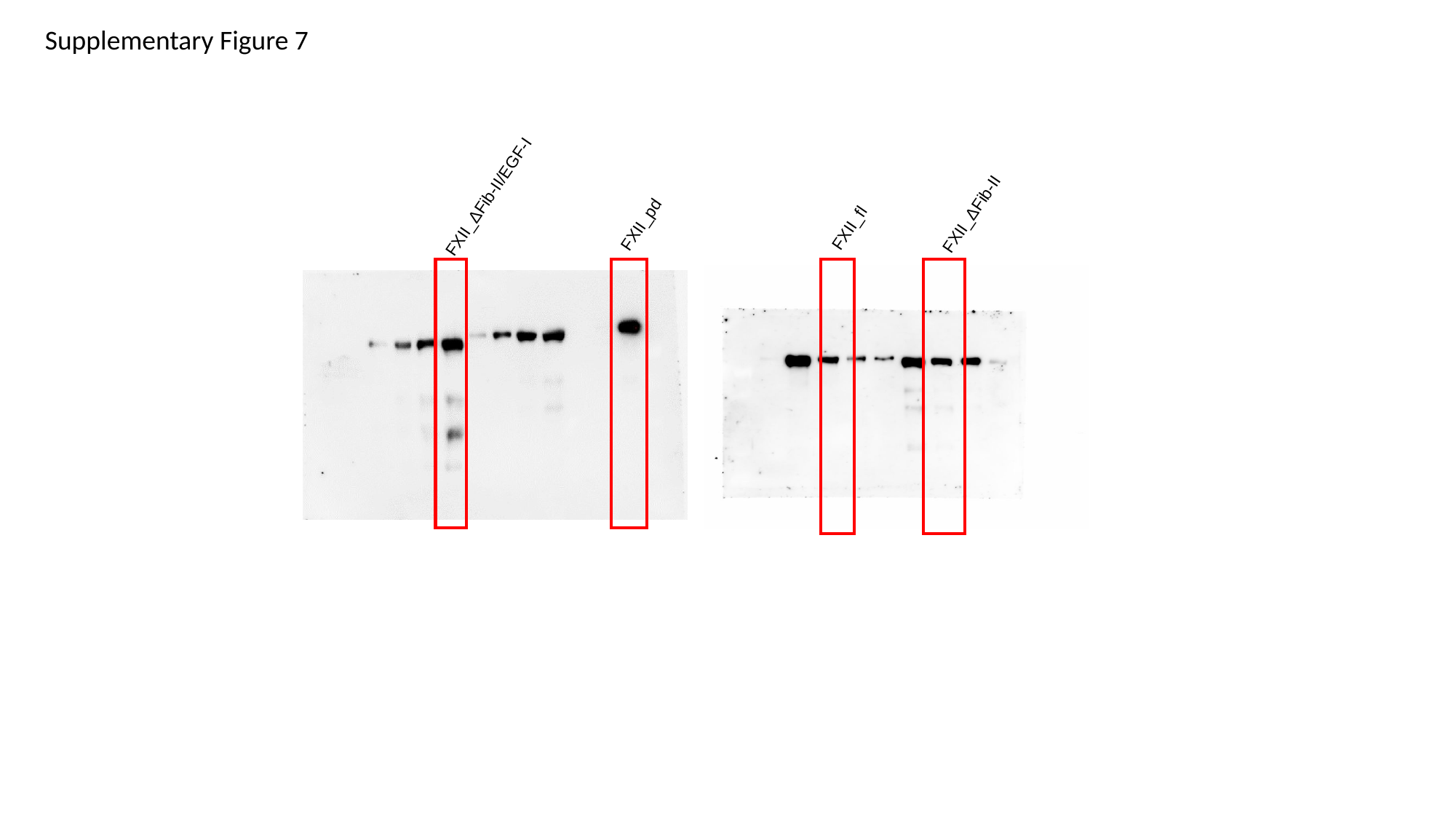

Supplementary Figure 7
FXII_fl
FXII_pd
FXII_ΔFib-II
FXII_ΔFib-II/EGF-I

Supplement: Supplementary file 4 — Source Data [file 41467_2021_25888_MOESM4_ESM.zip › SOURCE DATA/Supplementary Information/Source Data Supplementary Figure 7.pptx]

## Slide 1
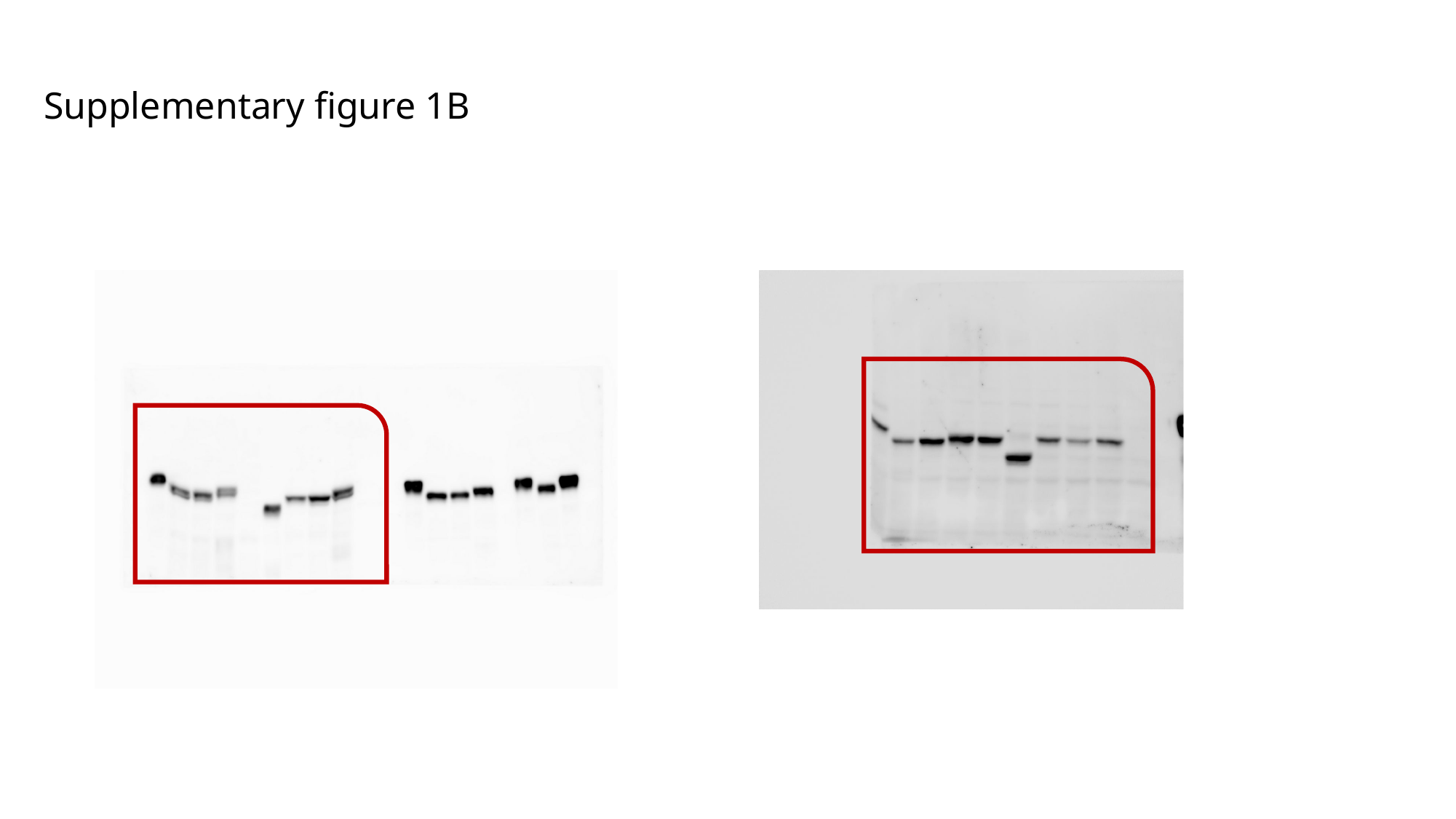

# Supplementary figure 1B

Supplement: Supplementary file 4 — Source Data [file 41467_2021_25888_MOESM4_ESM.zip › SOURCE DATA/Supplementary Information/Source Data Supplementary Figure 1B.pptx]

## Slide 1
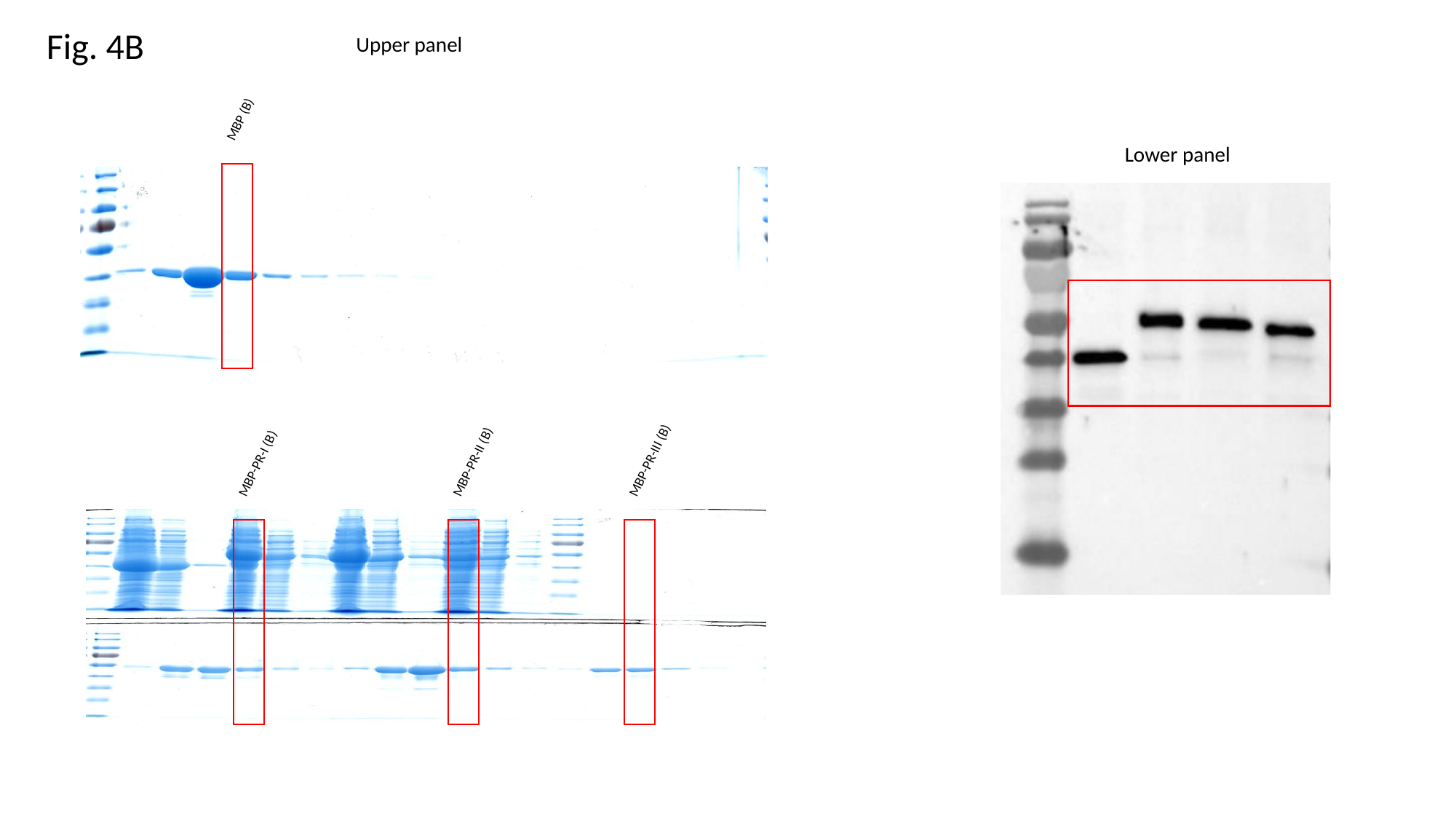

Upper panel
Fig. 4B
Lower panel
MBP (B)
MBP-PR-I (B)
MBP-PR-II (B)
MBP-PR-III (B)

Supplement: Supplementary file 4 — Source Data [file 41467_2021_25888_MOESM4_ESM.zip › SOURCE DATA/Main Manuscript/Source Data 4B_recFragments_western blot.pptx]

## Slide 1
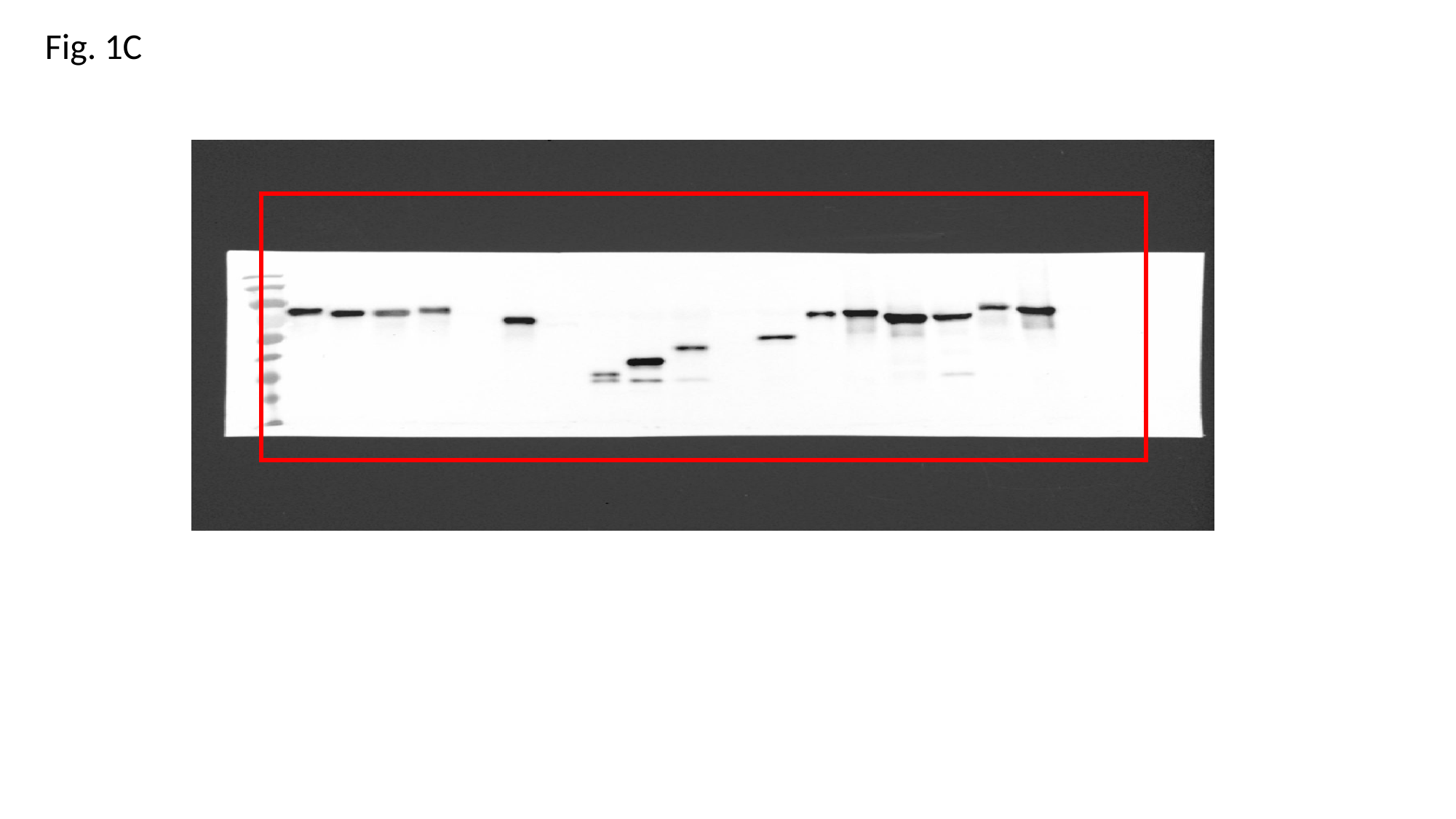

Fig. 1C

Supplement: Supplementary file 4 — Source Data [file 41467_2021_25888_MOESM4_ESM.zip › SOURCE DATA/Main Manuscript/Source Data 1C_extraExpression western blot.pptx]

## Slide 1
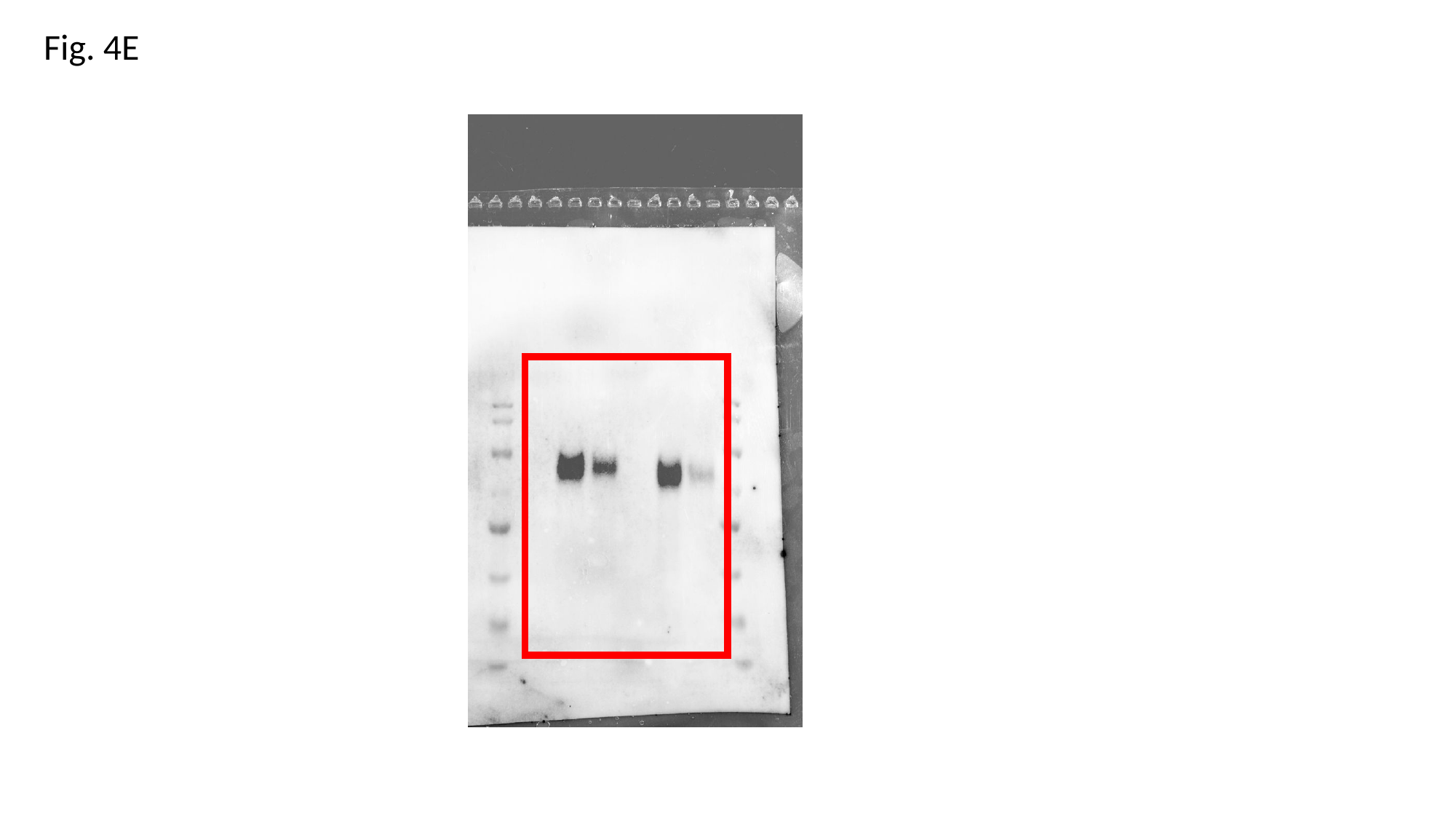

Fig. 4E

Supplement: Supplementary file 4 — Source Data [file 41467_2021_25888_MOESM4_ESM.zip › SOURCE DATA/Main Manuscript/Source Data 4F_Pullodown_western blot.pptx]

## Slide 1
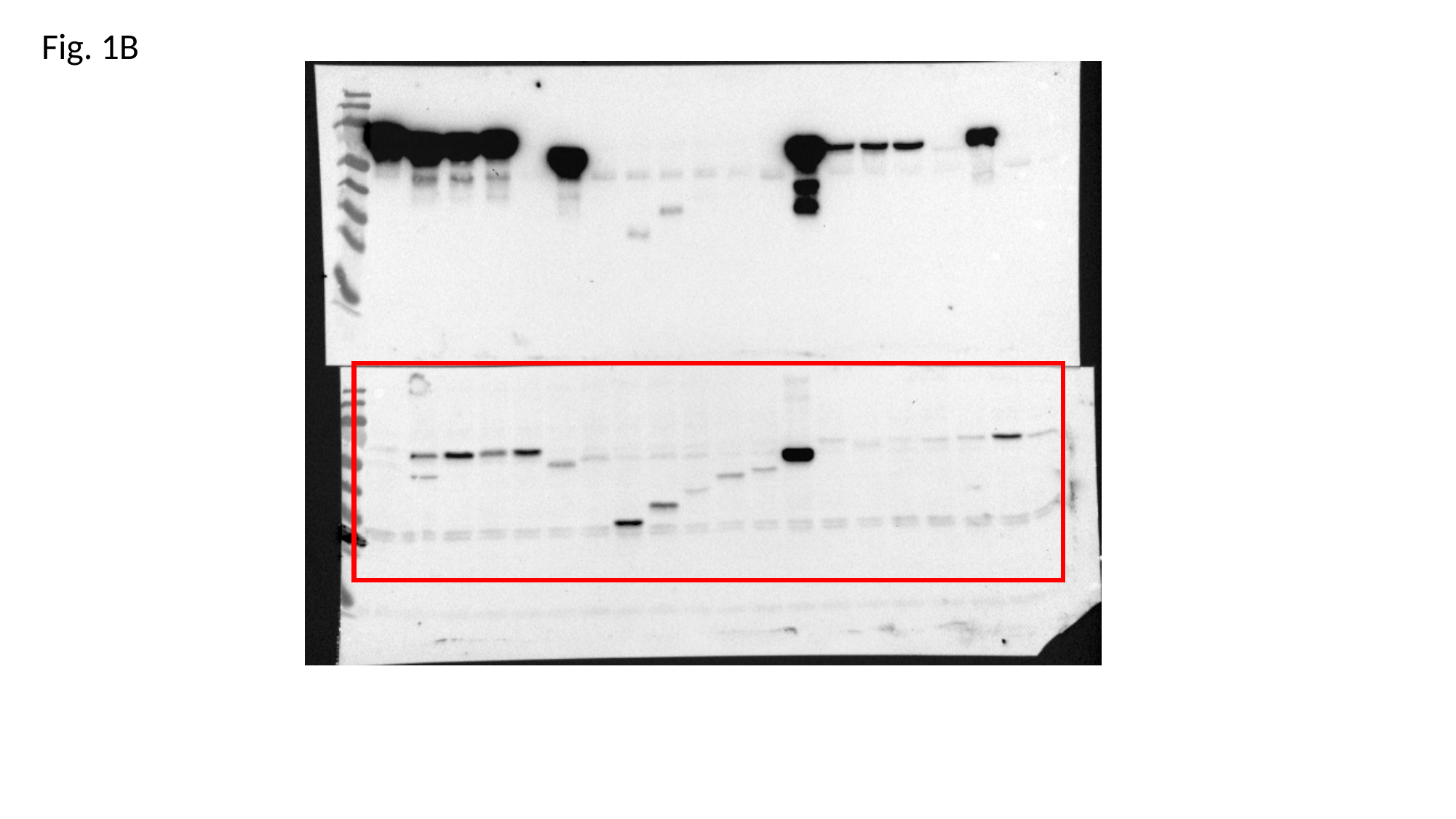

Fig. 1B

Supplement: Supplementary file 4 — Source Data [file 41467_2021_25888_MOESM4_ESM.zip › SOURCE DATA/Main Manuscript/Source Data 1B_intrExpression western blot.pptx]

## Slide 1
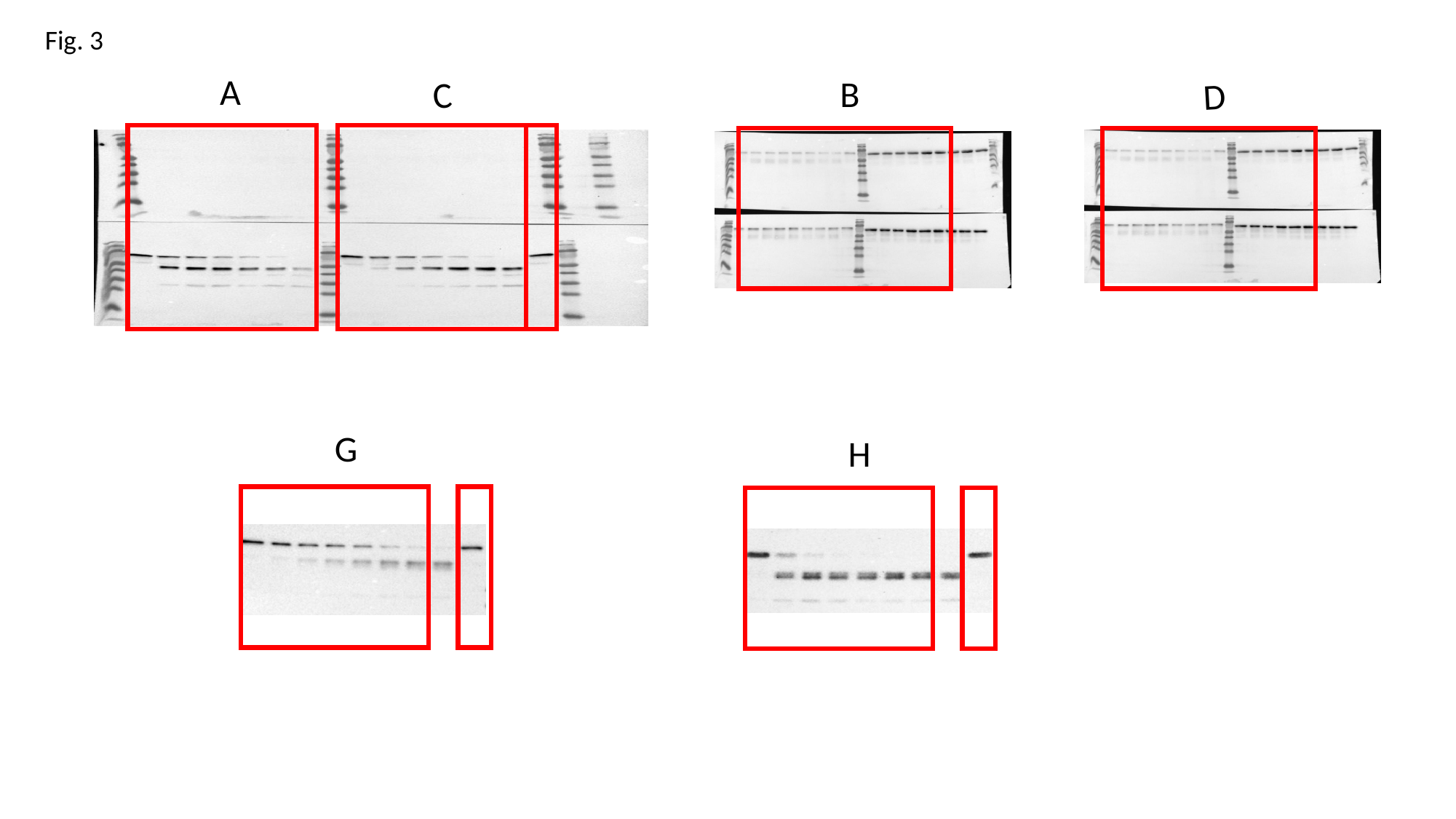

Fig. 3
 C
 A
 B
 D
 G
 H

## Slide 2
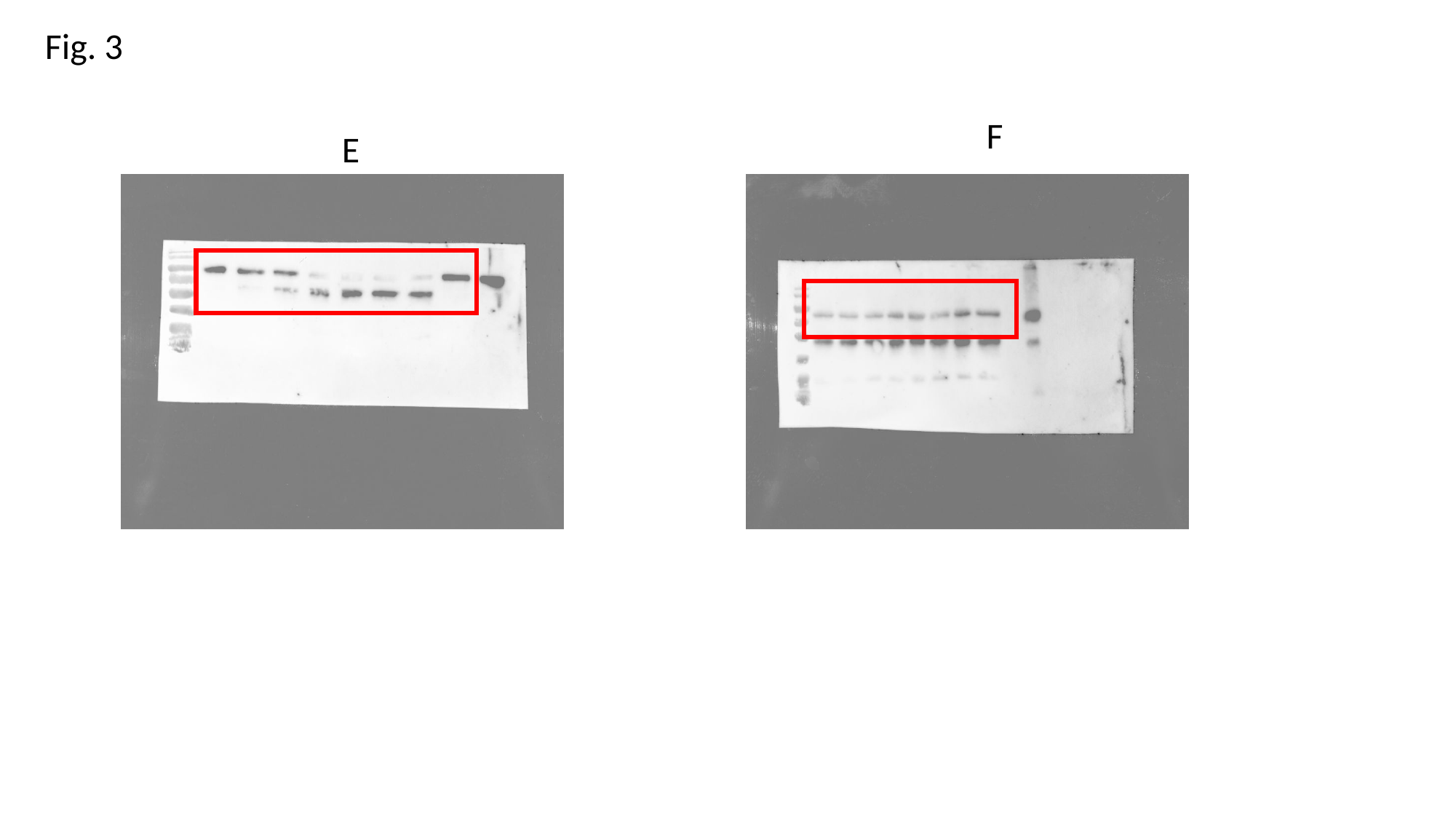

Fig. 3
 E
 F

Supplement: Supplementary file 4 — Source Data [file 41467_2021_25888_MOESM4_ESM.zip › SOURCE DATA/Main Manuscript/Source Data 3ABCDEFGH_cleavage western blot.pptx]

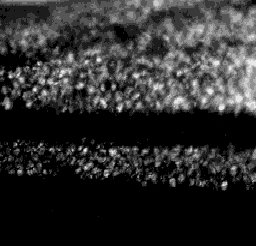

Supplement: Supplementary file 4 — Source Data [file 41467_2021_25888_MOESM4_ESM.zip › SOURCE DATA/Main Manuscript/Source Data 5A_Thrombosis_microscopeimages/FXII/FXII_40min.jpg]

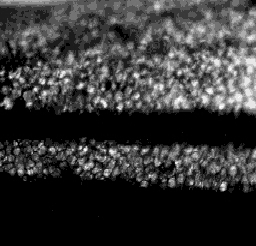

Supplement: Supplementary file 4 — Source Data [file 41467_2021_25888_MOESM4_ESM.zip › SOURCE DATA/Main Manuscript/Source Data 5A_Thrombosis_microscopeimages/FXII/FXII_20min.jpg]

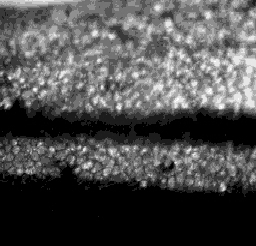

Supplement: Supplementary file 4 — Source Data [file 41467_2021_25888_MOESM4_ESM.zip › SOURCE DATA/Main Manuscript/Source Data 5A_Thrombosis_microscopeimages/FXII/FXII_0min.jpg]

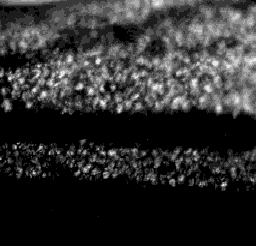

Supplement: Supplementary file 4 — Source Data [file 41467_2021_25888_MOESM4_ESM.zip › SOURCE DATA/Main Manuscript/Source Data 5A_Thrombosis_microscopeimages/FXII/FXII_10min.jpg]

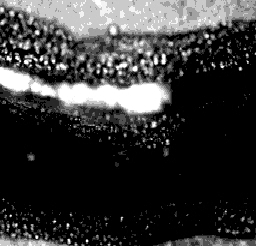

Supplement: Supplementary file 4 — Source Data [file 41467_2021_25888_MOESM4_ESM.zip › SOURCE DATA/Main Manuscript/Source Data 5A_Thrombosis_microscopeimages/FXII+FXIIwt/FXII+FXIIwt_40min.jpg]

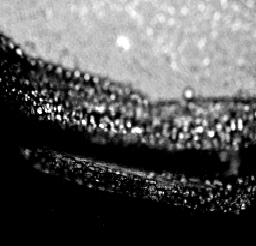

Supplement: Supplementary file 4 — Source Data [file 41467_2021_25888_MOESM4_ESM.zip › SOURCE DATA/Main Manuscript/Source Data 5A_Thrombosis_microscopeimages/FXII+FXIIwt/FXII+FXIIwt_0min.jpg]

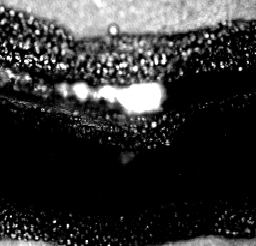

Supplement: Supplementary file 4 — Source Data [file 41467_2021_25888_MOESM4_ESM.zip › SOURCE DATA/Main Manuscript/Source Data 5A_Thrombosis_microscopeimages/FXII+FXIIwt/FXII+FXIIwt_20min.jpg]

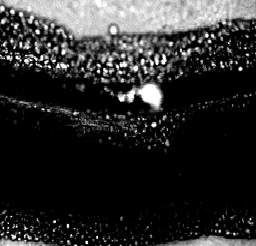

Supplement: Supplementary file 4 — Source Data [file 41467_2021_25888_MOESM4_ESM.zip › SOURCE DATA/Main Manuscript/Source Data 5A_Thrombosis_microscopeimages/FXII+FXIIwt/FXII+FXIIwt_10min.jpg]

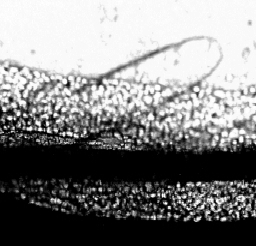

Supplement: Supplementary file 4 — Source Data [file 41467_2021_25888_MOESM4_ESM.zip › SOURCE DATA/Main Manuscript/Source Data 5A_Thrombosis_microscopeimages/FXII+FXIImut/FXII+FXIImut_40min.jpg]

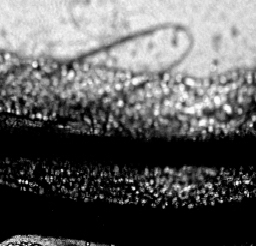

Supplement: Supplementary file 4 — Source Data [file 41467_2021_25888_MOESM4_ESM.zip › SOURCE DATA/Main Manuscript/Source Data 5A_Thrombosis_microscopeimages/FXII+FXIImut/FXII+FXIImut_20min.jpg]

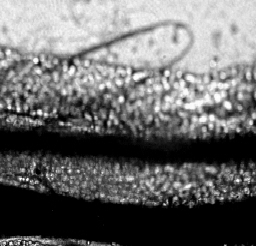

Supplement: Supplementary file 4 — Source Data [file 41467_2021_25888_MOESM4_ESM.zip › SOURCE DATA/Main Manuscript/Source Data 5A_Thrombosis_microscopeimages/FXII+FXIImut/FXII+FXIImut_10min.jpg]

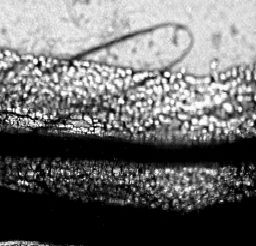

Supplement: Supplementary file 4 — Source Data [file 41467_2021_25888_MOESM4_ESM.zip › SOURCE DATA/Main Manuscript/Source Data 5A_Thrombosis_microscopeimages/FXII+FXIImut/FXII+FXIImut_0min.jpg]

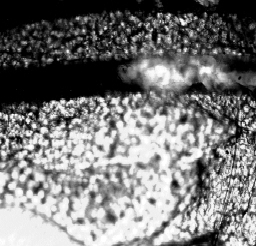

Supplement: Supplementary file 4 — Source Data [file 41467_2021_25888_MOESM4_ESM.zip › SOURCE DATA/Main Manuscript/Source Data 5A_Thrombosis_microscopeimages/WT/WT_40min.jpg]

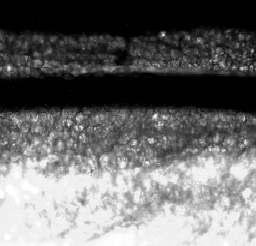

Supplement: Supplementary file 4 — Source Data [file 41467_2021_25888_MOESM4_ESM.zip › SOURCE DATA/Main Manuscript/Source Data 5A_Thrombosis_microscopeimages/WT/WT_0min.jpg]

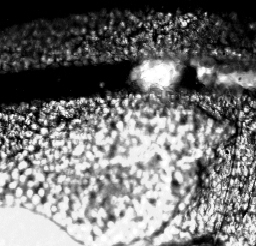

Supplement: Supplementary file 4 — Source Data [file 41467_2021_25888_MOESM4_ESM.zip › SOURCE DATA/Main Manuscript/Source Data 5A_Thrombosis_microscopeimages/WT/WT_20min.jpg]

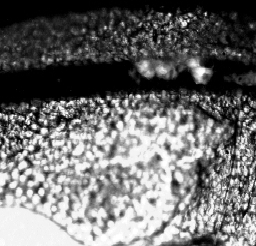

Supplement: Supplementary file 4 — Source Data [file 41467_2021_25888_MOESM4_ESM.zip › SOURCE DATA/Main Manuscript/Source Data 5A_Thrombosis_microscopeimages/WT/WT_10min.jpg]
